# Outside-in signaling through the major histocompatibility complex class-I cytoplasmic tail modulates glutamate receptor expression in neurons

**DOI:** 10.1038/s41598-023-38663-z

**Published:** 2023-08-11

**Authors:** Brett A. Eyford, Maciej J. Lazarczyk, Kyung Bok Choi, Merina Varghese, Hitesh Arora, Suresh Kari, Lonna Munro, Cheryl G. Pfeifer, Allison Sowa, Daniel R. Dickstein, Dara L. Dickstein, Wilfred A. Jefferies

**Affiliations:** 1 Michael Smith Laboratories, The University of British Columbia, 2185 East Mall, Vancouver, BC V6T 1Z4 Canada; 2 The Vancouver Prostate Centre, Jack Bell Research Centre, 2660 Oak Street, Vancouver, BC V6H 3Z6 Canada; 3 Centre for Blood Research, University of British Columbia, 2350 Health Sciences Mall, Vancouver, BC V6T 1Z3 Canada; 4 Djavad Mowafaghian Centre for Brain Health, University of British Columbia, 2215 Wesbrook Mall, Vancouver, BC V6T 2B5 Canada; 5 Division of Institutional Measures, Department of Medical Direction and Quality, University Hospitals of Geneva, Geneva, Switzerland; 6 Nash Family Department of Neuroscience, Icahn School of Medicine at Mount Sinai, 1425 Madison Ave, New York, NY 10029 USA; 7 Department of Microbiology and Immunology, University of British Columbia, 1365-2350 Health Sciences Mall, Vancouver, BC V6T 1Z3 Canada; 8 Department of Zoology, University of British Columbia, 2370-6270 University Blvd., Vancouver, BC V6T 1Z4 Canada; 9 Department of Medical Genetics, University of British Columbia, 1364-2350 Health Sciences Mall, Vancouver, BC V6T 1Z3 Canada; 10 Department of Pathology, Uniformed Services University of Health Sciences, 4301 Jones Bridge Road, Bethesda, MD 20814 USA; 11 The Henry M. Jackson Foundation for the Advancement of Military Medicine (HJF), 6720A Rockledge Drive, Bethesda, MD 20817 USA

**Keywords:** Biochemistry, Immunology

## Abstract

The interplay between AMPA-type glutamate receptors (AMPARs) and major histocompatibility complex class I (MHC-I) proteins in regulating synaptic signaling is a crucial aspect of central nervous system (CNS) function. In this study, we investigate the significance of the cytoplasmic tail of MHC-I in synaptic signaling within the CNS and its impact on the modulation of synaptic glutamate receptor expression. Specifically, we focus on the Y321 to F substitution (Y321F) within the conserved cytoplasmic tyrosine YXXΦ motif, known for its dual role in endocytosis and cellular signaling of MHC-I. Our findings reveal that the Y321F substitution influences the expression of AMPAR subunits GluA2/3 and leads to alterations in the phosphorylation of key kinases, including Fyn, Lyn, p38, ERK1/2, JNK1/2/3, and p70 S6 kinase. These data illuminate the crucial role of MHC-I in AMPAR function and present a novel mechanism by which MHC-I integrates extracellular cues to modulate synaptic plasticity in neurons, which ultimately underpins learning and memory.

## Introduction

In an unexpected biological twist, major histocompatibility complex class I (MHC-I) molecules have a dual role entwining the immune system with the central nervous system (CNS). In the brain, MHC-I is expressed in neurons under regular physiological conditions^1–4^. MHC-I plays a role in dendrite morphogenesis and synapse pruning, functions that are considered autonomous from its role in the immune system^2,5,6^. Indeed, synaptic density was seen to increase when stable surface MHC-I is absent^2,7–10^, causing impairment in memory and learning^11–14^. Interestingly, the impact of MHC-I on synaptic density and plasticity, initially thought to occur at early neurodevelopmental stages^1,2^, can persist throughout life or may re-emerge at an advanced age^10^.

The exact mechanism of MHC-I activity and signalling in the CNS remains elusive and has only been studied in the context of genetic loss of MHC-I and associated molecules with the goal of identifying the genes involved in synaptic plasticity. One hypothesis speculates that MHC-I acts in trans by binding putative partner proteins across the synaptic cleft. Indeed, the CD3ζ and TCRβ components of the T-cell receptor, which is the most common MHC-I partner, are expressed in neurons in the CNS^15,16^. Other known MHC-I binding proteins, such as paired immunoglobulin-like receptor B and Ly49, are also expressed in the CNS and are thought to interact with MHC-I at the synapse^17,18^. However, mice deficient in these molecules do not recapitulate the phenotype of MHC-I knockout mice^11,19^, suggesting that MHC-I acts in *cis*. Indeed, MHC-I mediates outside-in or reverse signalling via its intracellular domain^20^.

Our previous work identified a highly conserved tyrosine residue in the cytoplasmic domain of MHC-I that is required for antigen presentation of exogenously-acquired, but not endogenously-synthesized, antigens in dendritic cells (DCs) to CD8+ Cytotoxic T Lymphocytes (CTLs)^21^. In a mouse model, the conserved tyrosine residue (Y_321_) of MHC-I H-2K^b^ was substituted with phenylalanine (termed ΔY). The resultant ΔY molecules have an impaired ability to internalize from the DC cell surface and cannot traffic to the endolysosomal (LAMP-1 positive) compartments^22^. As a functional consequence, DCs derived from ΔY transgenic mice are impaired in their ability to cross-present exogenous sourced protein antigens^23^. In addition, ΔY mice mount inferior CTL responses in vivo upon infection with two viruses that can cause respiratory illnesses^23^, vesicular stomatitis virus (VSV) and heat-killed Sendai virus (a classic viral source of exogenous antigen), as well as the intracellular bacteria, *Listeria monocytogenes* that can cause pneumonia^24^. The Y321F mutation appears to specifically abrogate cross-presentation, as ΔY DCs are able to efficiently present endogenously-produced protein antigens to specific CTLs in vitro^23^.

The conserved Y_321_ residue is part of a conserved YXXA sequence that is highly reminiscent of tyrosine-based YXXØ endocytic motifs found in other transmembrane proteins (Y is tyrosine, X is any amino acid, A is alanine, and Ø is a bulky, hydrophobic amino acid). Thus, YXXA constitutes an endocytic sorting motif directly responsible for the trafficking of MHC- I through endocytic antigen-loading compartments and ultimately essential for MHC-I antigen cross-presentation and for MHC-I recycling to the cell surface via tyrosine phosphorylation^21^. This is not unique to murine cells as MHC-I has also been observed within endocytic compartments of human DCs, where they acquire exogenous antigens for cross-presentation. The human MHC-I equivalents, human leukocyte antigen-A (HLA-A) and B (HLA-B) molecules, both contain homologous YXXA motifs, suggesting that endocytic trafficking is controlled by similar mechanisms to those used by murine MHC-I. HLA-C lacks the conserved tyrosine residue found in most MHC-I cytoplasmic domains, but trafficking of this molecule is likely to be dependent upon a unique downstream dileucine motif^21^. Therefore, MHC-I molecules recycled from the plasma membrane are a source of loadable MHC-I for participation in antigen cross-presentation^22,23^. Finally, the cytoplasmic tail of MHC-I also appears to play a role in repressing Toll-like receptor-mediated inflammation^20^ and other physiological functions^25^, but no functional studies have been implemented that address its role in the CNS nor the signaling and cellular mechanisms involved with synaptic plasticity.

We recently discovered that the cytoplasmic tail of MHC-I proteins regulates spine and synapse density and morphology in hippocampal neurons. We demonstrated that MHC-I in the CNS functions in *cis* to mediate outside-in function via its intracellular domain, ultimately facilitating synaptic plasticity. In the present study, we aim to provide mechanistic details that could connect MHC-I to its function in neurons. To ascertain the role of the MHC-I cytoplasmic tail in excitatory synaptic function and associated intracellular signalling in the CNS, we examined pre- and post-synaptic protein expression and signaling through kinase activity in hippocampal neurons from a mouse strain expressing a Y321 to F substitution (Y321F) of the conserved cytoplasmic Wild-type (WT) tyrosine (Y321) in the YXXΦ motif of the cytoplasmic tail of MHC-I in animals lacking all other MHC-I molecules.

These experiments thus reveal a novel mechanism of regulation of excitatory signalling in the hippocampus involving MHC-I sensing the external environment to convey information through its cytoplasmic tyrosine-based motif.

## Results

### The mutated YXXΦ motif alters the level of expression of AMPA receptors at the synapse

**Figure 1:**
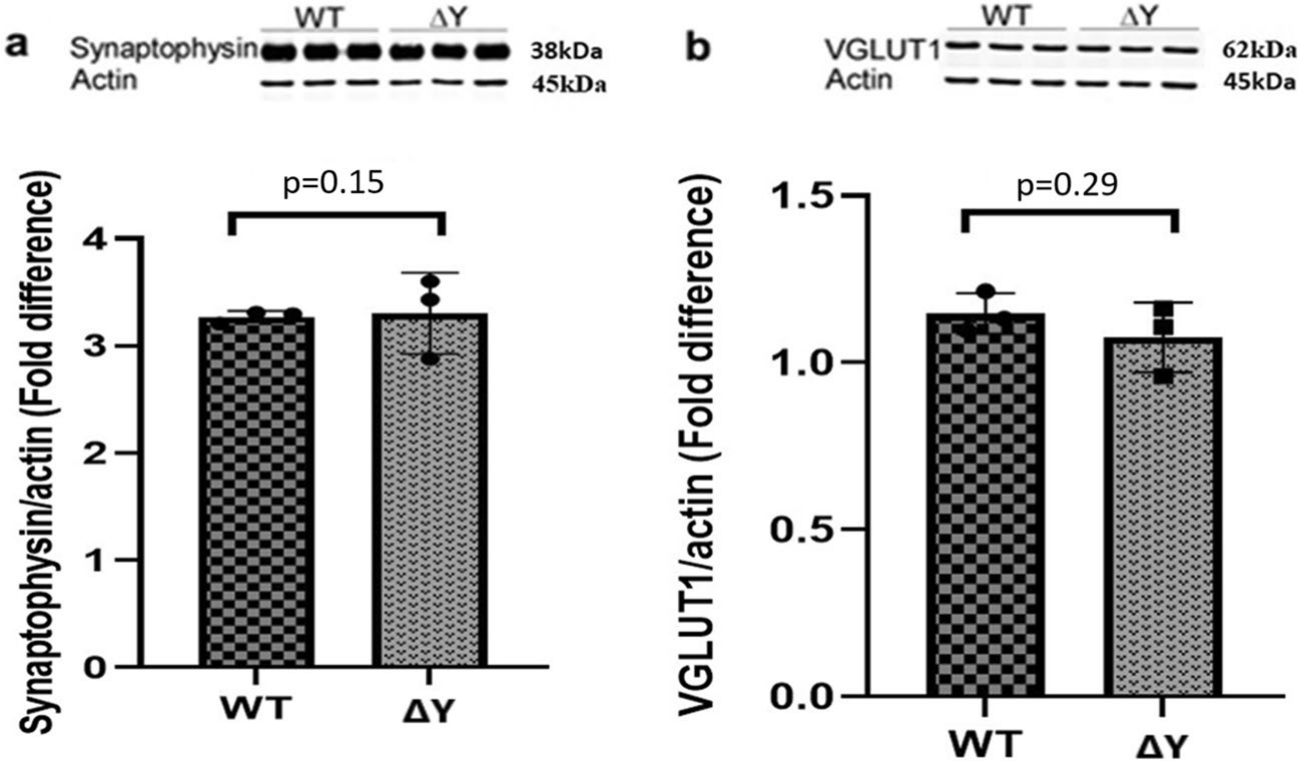
The ΔY mutation has no effect on the expression of pre-synaptic proteins in the hippocampus. Representative Western blots and analysis of integrated fluorescent intensities of pre-synaptic proteins, synaptophysin and VGlut1, from WT and ΔY mice, n = 3 independent experiments. Each lane for the Western blots represents a different mouse. The data was pooled from each group in the graphs, and statistical analysis was performed between each group. All proteins were normalized to actin, and values represent means ± SEM. (**a**) Synaptophysin has an apparent molecular weight of 38 kDa, and beta-actin has an apparent molecular weight of 45 kDa. There is no statistical difference between protein expression in VGlut1 expression in ΔY compared to WT mice (p = 0.29). (**b**) Based on the standard of molecular weight (MW) markers proteins, VGLUT1 has apparent molecular weight of 62 kDa. There is no statistical difference between protein expression in synaptophysin expression in ΔY compared to WT mice (p = 0.15) using an unpaired *t* test. The full-gels and their analysis are provided in the Supplementary figures, including the migration of each protein relative the MW ladder.

We previously observed differences in the spine and synaptic densities and memory in a mouse strain expressing a Y321 to F substitution (Y321F) of the conserved cytoplasmic WT tyrosine (Y321) in the YXXΦ motif of the cytoplasmic tail of MHC-I in animals lacking all other MHC-I molecules^26^. Here, for simplicity we abbreviate Y321F substitution to ΔY and sought to establish a molecular mechanism to explain our previous observations^26^. Initially, we examined whether the changes we observed between WT and ΔY mice were associated with alterations in synaptic protein expression. Western blot analysis of pre-synaptic proteins synaptophysin in Fig. 1a (p = 0.15) and vesicular glutamate transporter 1 (VGluT)1 in Fig. 1b (p = 0.29) in the hippocampus showed no statistical differences between genotypes.

**Figure 2:**
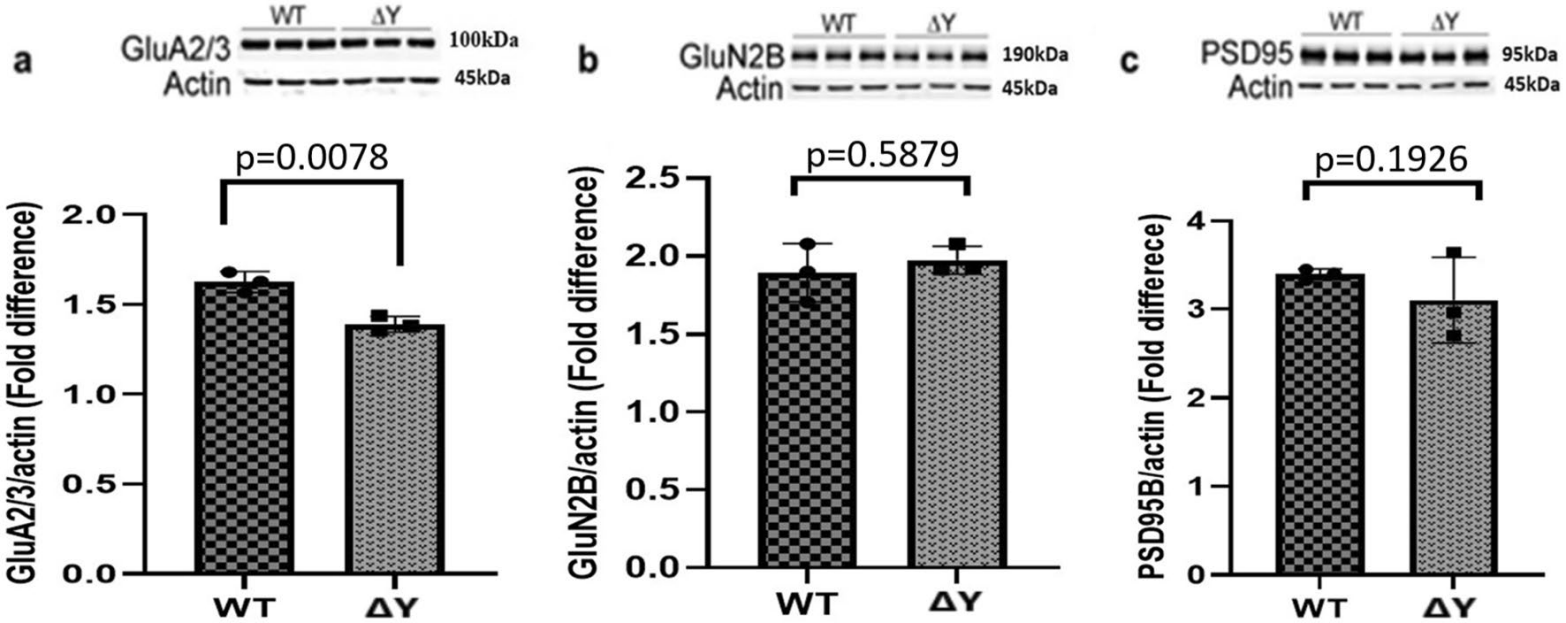
The ΔY mutation reduces the expression of AMPA receptors. Representative Western blots and analysis of integrated fluorescent intensities of post-synaptic proteins GluA2/3, GluN2B, and PSD95 in ΔY and WT mice, n = 3. (**a**) There was an observed significant decrease in GluA2/3 in ΔY mice compared to WT mice (p = 0.0078) using an unpaired *t* test. (**b**) No change in GluN2B in ΔY mice compared to WT mice (p = 0.5879) using an unpaired *t* test. (**c**) No changes in PSD95 expression in ΔY mice compared to WT mice (p = 0.1926) using an unpaired *t* test*.* All proteins were normalized to beta-actin and values represent means ± SEM. Each lane for the western blots represents a different mouse. The data was pooled from each group in the graphs, and statistical analysis was performed between each group. Based on the standard of molecular weight markers, GluA2/3 has an apparent molecular weight of 100 kDa, GluN2b has an apparent molecular weight of 190 kDa, PSD95 has an apparent molecular weight of 95 kDa, and actin has an apparent molecular weight of 45 kDa. The full-gels and their analysis is provided in the Supplementary figures, including the migration of each protein relative to the MW ladder.

We also analyzed levels of three post-synaptic proteins: postsynaptic density protein 95 (PSD95), the *N*-Methyl-d-aspartate (NMDA) receptor GluN2B subunit, and the α-Amino-3-hydroxy-5-methyl-4-isoxazolepropionic acid (AMPA) receptor GluA2/3 subunit in hippocampal homogenates. We did note a significant decrease in GluA2/3 expression in ΔY mice in comparison to WT littermates in Fig. 2a (p = 0.0078) and no change in GluN2B in Fig. 2b (p = 0.5879) or PSD95 expression in Fig. 2c (p = 0.1926).

### The ΔY mutation alters the phosphorylation of various kinases and transcription factors

**Figure 3:**
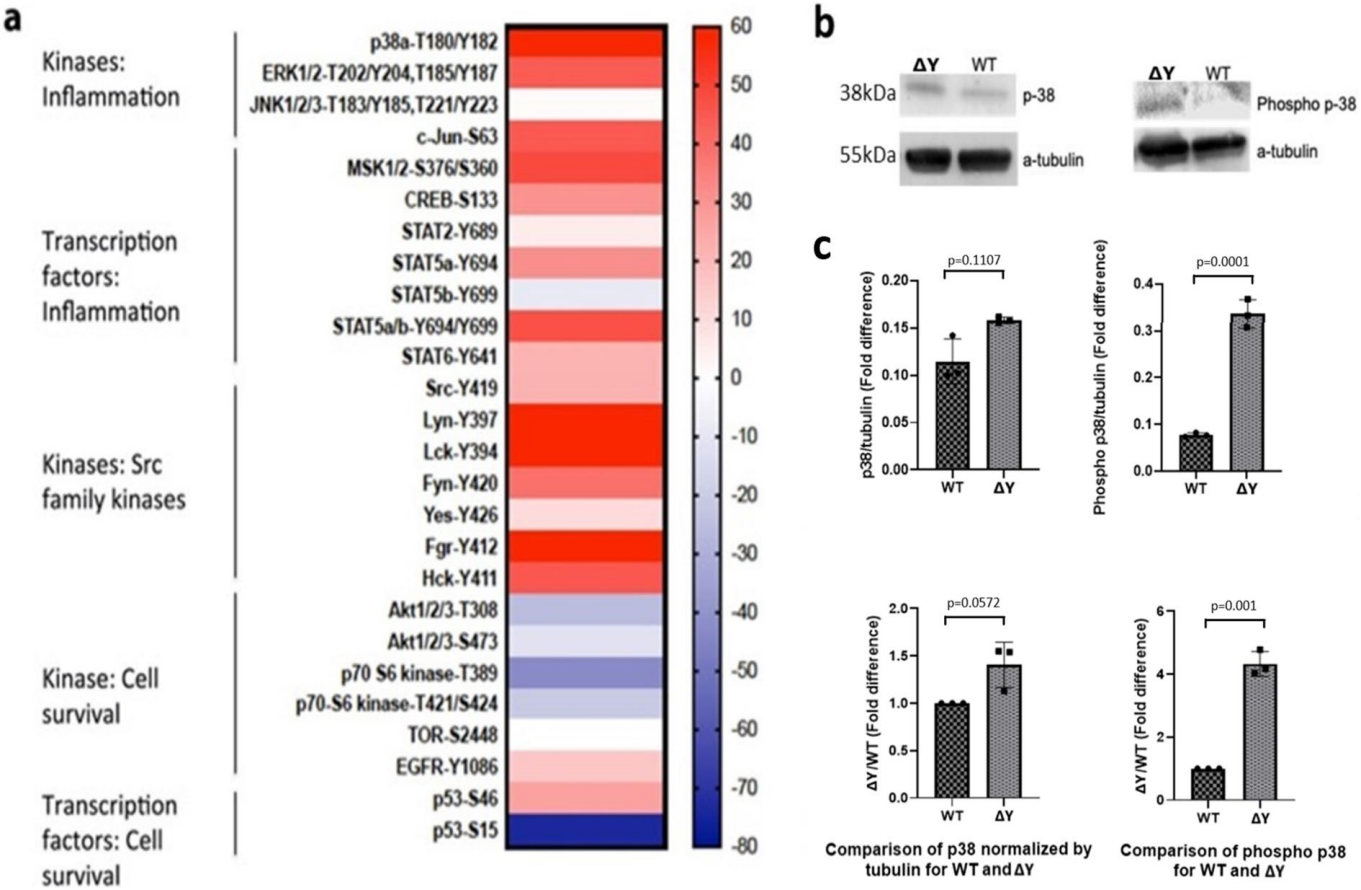
The ΔY mutation alters phosphorylation of various kinases and transcription factors. (**a**) Relative changes in protein kinase phosphorylation levels of target proteins in brain tissue lysates from ΔY and WT mice represented in a heat map as a ratio of the ΔY/WT phosphokinase levels. Increased phosphorylation was observed in various kinases and transcription factors involved in inflammation and members of the Src family kinases with roles in neuronal and synaptic plasticity, while decreased phosphorylation was observed in kinases involved in cell survival. Kinase phosphoarray assays were performed in two animals and analyzed in duplicate in each kinase assay before establishing the means that are shown in the heatmap. (**b**) Validation of the protein phosphoarray by western blot analysis for p38a T180/Y182. Based on the standard molecular weight markers, the control alpha-tubulin has an apparent molecular weight of 55 kDa, and p38 has an apparent molecular weight of 38 kDa. (**c**) Expression levels of phosphorylated and non-phosphorylated p38 in brain tissue lysates from ΔY and WT mice, represented as mean ± SEM. All comparisons were analyzed using a paired *t* test where p < 0.05 was consider significant. The antibodies used were Anti-p38 alpha/MAPK14 antibody, Anti-p38 (phospho T180 + Y182) antibody and Anti-alpha Tubulin antibody). Secondary antibodies: Goat Anti-Mouse IgG H&L (HRP) preabsorbed (ab97040) and Invitrogen. Goat anti-Rabbit IgG (H + L) Secondary Antibody, HRP (31460) The full-gels and their analysis is provided in the Supplementary figures, including the migration of each protein relative the MW ladder.

Given the dual-functional role of the YXXΦ motif in both endocytosis and cellular signalling^20^, we performed an assay to determine which signalling pathways were altered in the ΔY mice as indicated by differential phosphorylation levels of kinases and transcription factors. We found that many kinases and transcription factors were differentially phosphorylated in ΔY mice, as compared to WT controls (Fig. 3a). Phosphorylation of mitogen-activated protein kinases (MAPKs) involved in inflammation was drastically up-regulated. In particular, phosphorylation of p38α and extracellular signaling regulated kinase 1 (ERK1), which are downstream of MAPK, were up-regulated by 60 and 40 fold respectively. Downstream transcription factors like the immediate early gene c-Jun or transcription factor AP-1, the serine-threonine protein kinase MSK1 or ribosomal protein S6 kinase alpha-5, cyclic AMP-responsive element-binding protein (CREB) and signal transducer and activator of transcription 5A (STAT5a) also showed a drastic increase in their phosphorylation levels, but the increase in levels of STAT2 and STAT6 was not that profound. Non-receptor tyrosine-protein kinases such as Lyn, Lck, Fgr and Fyn of the Src family kinases (SFKs) showed up to a 50-fold up-regulation. In contrast, kinases involved in cell survival such as RAC-alpha serine/threonine-protein kinase or Akt1 and ribosomal protein S6 kinase beta-1 or p70S6 kinase exhibited a decrease in phosphorylation. Serine/threonine-protein kinase mechanistic/mammalian target of rapamycin (mTOR) levels remained unchanged and levels of the receptor tyrosine kinase epidermal growth factor receptor (EGFR) increased slightly. Phosphorylation of transcription factor p53 at serine 46 was slightly up-regulated and at serine 15 was 80-fold down-regulated (Fig. 3a). Evidence exists that the p38 MAP kinase pathway dysregulation of MHC-I-expressing monocytic cells correlates with MHC-I misfolding^27^. Validating the results in the protein array, the phosphorylation levels of p38 in the brain homogenates of ΔY mice was up-regulated compared to WT and representative western blots are shown in Fig. 3a. Results summarizing the averages of 3 experiments are shown in Fig. 3c. There was no significant difference in p38 protein expression between brain tissue lysates from ΔY and WT mice normalized to tubulin (p = 0.1107), nor as a ratio between ΔY and WT mice (p = 0.0572). However, a significant difference in phospho-p38 levels normalized to tubulin (p = 0.0001) and as a ratio between ΔY and WT mice (p = 0.001) was clearly evident. Overall, the validation studies in Fig. 3b,c are consistent with the phosphoprotein arrays in Fig. 3a showing the phosphorylation of p38 in ΔY mice is statistically greater than in WT brain extracts.

**Figure 4:**
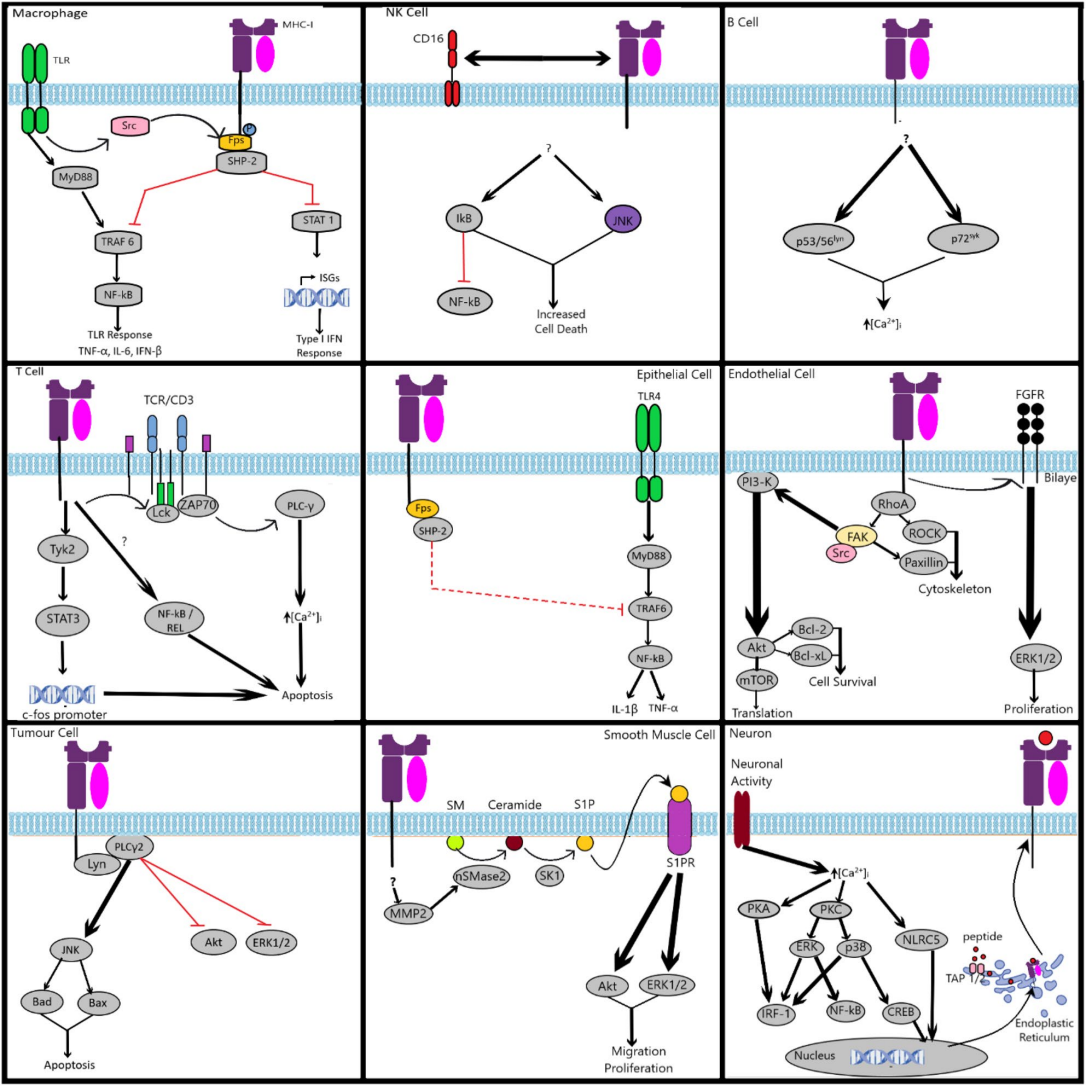
Summary of reverse MHC-I signalling pathways in various cell types. Summary of the main reverse (inside-out) MHC-I signalling pathways in immune and non-immune cells. A more detailed description can be found in a review by Valenzuela and Reed^28^ as well as Muntjewerff et al.^25^.

Figure 4 illustrates the major MHC-I signalling pathways that have been established in various immune and non-immune cell types.

## Discussion

The mechanisms by which the cytoplasmic tail of MHC-I plays a role in the CNS, in particular how it regulates synaptic function, remain unknown. In the periphery, the cytoplasmic domain of MHC-I is known to be involved in the regulation of signalling events, specifically in the phosphorylation of specific kinases. Furthermore, there is no single or unique disease pathway that these kinases are involved in; on the contrary, the same kinases can be involved in normal cell physiology and signalling across multiple organ systems, including the cardiac, pulmonary, central and peripheral nervous system, as well as in pathological cell functioning associated with multiple diseases and disorders, including cancers, cardiac, pulmonary, neurology, and neurodegeneration, all of which are known to involve one or more of these kinases. However, based on the kinase array data, we have identified putative mechanisms by which this regulation of CNS functions can occur. It is known that the phosphorylation of the Y321 residue on MHC-I results in the recruitment of kinases for intracellular signalling^29,30^. The kinase array was chosen because it covers kinases involved in oncology, cardiology, pulmonology, and neurobiology and that are considered important in normal cell physiology and disease. This array data revealed a variety of proteins that were differentially phosphorylated in ΔY mice. In particular, we observed changes in proteins implicated in inflammation, survival and growth, such as p38 and ERK1/2, Src, Fyn, Lyn, Lck, Fgr, p70S6 and p53. Of these proteins, we observed increases in the phosphorylation of many members of the Src family kinases (SFK) like Fyn or Lck. In the CNS, SFKs, such as Fyn, Lyn, Lck and Fgr are expressed during development and are implicated in proliferation and differentiation. However, SFKs are also expressed in differentiated neurons in the adult CNS where they regulate ion channel activity, such as NMDA receptors (NMDARs), and synaptic transmission at glutamatergic synapses^31^. Moreover, PSD and SFKs are components of the NMDAR complex (reviewed in^32^). This expression pattern and change in function may correspond to MHC-I function as our previous study demonstrated that MHC-I also plays a role in the adult CNS^10^. Indeed, Fyn kinase that is hyperphosphorylated is known to play a crucial role in synapse development^33^, myelination^34^, long-term potentiation^35,36^, learning and memory^37–39^, and NMDA receptor activity^40^. Interestingly, spine density in the hippocampus, while unaltered in young Fyn-deficient mice, is significantly decreased in aged Fyn^−/−^ animals^41^. Out of necessity our samples were frozen for storage and then thawed before kinase assays were performed.

Previously, Fyn knockout mice were shown to exhibit reduced axonal branching in the cerebellar cortex^42^, decreased spine density in layer V pyramidal neurons^41^, as well as shortages in long-term spatial and potentiation learning^35^, underscoring their functional role in the dendritic compartment. For Fyn to be able to target the dendritic compartment efficiently, it requires the assistance of Tau, a microtubule-associated protein that has been found to be associated with neurodegenerative diseases such as Alzheimer's disease^43,44^, a process that is aided by interactions of the SH3 domain of Fyn with Tau, as well as the interaction of the SH2 domain of Fyn with tyrosine-phosphorylated Tau^45,46^. Additionally, the synaptic localization of Tau, as well as Fyn, has been associated with the increased expression of Tau, especially mutant forms of it such as P301L (found in frontotemporal dementia)^43,44,47^. This topic has been extensively covered by Padmanabhan et al.^48^.

Another mechanism whereby synaptic ultrastructure is modulated involves the expression of the AMPA receptor GluA2/3. We have previously shown that the complete absence of MHC-I results in increased expression of GluN2B but no change in GluA2/3 expression^10^. Our finding of decreased GluA2/3 in ΔY MHC-I mice implies that the cytoplasmic domain of MHC-I is involved in the expression of GluA2/3, but not GluN2B, a subunit of the respective receptors. Trafficking of GluA2/3 subunits to the synapse is constitutive and not activity-dependent^49^, and its impact on spine growth and synapse stability is quite complex. Overexpression of GluA2 increases spine length, width and density in hippocampal neurons in vitro^50^. Meanwhile, in vitro knockdown of this receptor subunit results in smaller and fewer spines in hippocampal neurons, whereas in vivo knockout results in fewer mushroom spines but more thin spines in the dentate gyrus^50,51^. These reports are in agreement with the decreased GluA2/3 expression and increased thin spines we observed in the hippocampus of ΔY MHC-I mice^50,51^.

Many intracellular signalling molecules are involved in the regulation of AMPAR expression at the synapse. Some of these proteins include the kinases and transcription factors detected in our array data, such as Lyn, which has been associated previously with AMPARs. In addition to being a ligand-gated ion channel, AMPARs also act as cell-surface signal transducers due to their interaction with Lyn kinase^52^. This interaction facilitates the activation of the MAPK signalling pathway, which includes ERK1/2 and p38, and may contribute to synaptic plasticity and neuroprotection by increasing the expression of neurotrophic factors such as brain-derived neurotrophic factor^52,53^. By interacting with AMPARs, Lyn kinase phosphorylates the tyrosine residue in the GluA2 subunit of the AMPAR near the C-terminus and regulates AMPAR receptor surface expression and synaptic targeting of GluA2^54^. In our model, we observed a decrease in GluA2/3 expression in ΔY mice and an up-regulation of phosphorylation of Lyn kinase. This follows the relationship above such that as GluA2/3 expression decreases, there is less protein available for Lyn to bind to and become activated, leading to more phosphorylated Lyn kinase and less recycling of GluA2/3^54,55^. Of the kinases with decreased levels of phosphorylation, p70S6 kinase is a member of the ribosomal s6 kinase family of serine/threonine kinases. In the CNS, it is possible that p70S6 kinase can have an effect on GluA2 expression and neuronal complexity via mTOR, a positive regulator of GluA2 synthesis (reviewed in^56^). A decrease in GluA2/3 via the inhibition of mTOR and p70S6 activity has been implicated in controlling dendritic morphology^57^ and synaptic plasticity^58,59^. It is possible that the YXXΦ motif in the cytoplasmic tail of MHC-I may be involved in this pathway, given the decrease in GluA2/3 and the decrease in the phosphorylation of p70S6 kinase observed in our study.

Further, recently we demonstrated that the cytoplasmic tail of MHC-I proteins regulates spine and synapse density and morphology in hippocampal neurons, suggesting that synaptic plasticity is facilitated by MHC-I in the CNS functioning in *cis* to mediate outside-in function via its intracellular domain^26^. It is interesting that there are other existing examples of non-immune cells found to contribute to inflammation promoted by reversing MHC-I signal transduction. For example, bronchiolitis obliterans syndrome develops in patients that have received a lung transplant due to the development of anti-HLA antibodies, which is caused by the activation of airway epithelial cells by HLA-A antibodies^60^. This is accompanied by the production of growth factors (PDGF, HB-EGF, IGF-1, and bFGF), which lead to the proliferation of the fibroblasts, explaining the processes of tissue remodelling and formation of fibrous tissue observed in this phenomenon^60^. Similarly MHC-I outside-in signaling occurs in macrophages where MHC-I was proposed to modulate TLR signaling in epithelial cells, leading to an increase of pro-inflammatory cytokines such as IL-1β and TNF-α^61^. In addition, Wu et al.^61^ were able to demonstrate that MHC-I cross-linking enhanced the interaction between MHC-I and Fps, while Fps knockdown resulted in decreased levels of SHP-2 and increased cytokine production, indicating that MHC-I on endometrial cells negatively regulates TLR-4-induced inflammatory response by enhancing the Fps-SHP-2 pathway. Thus, outside-in signaling through the MHC-I cytoplasmic domain may have wider impact on physiology than previously considered.

Overall, the present study identifies a mechanism in neurons that allows MHC-I to integrate extracellular cues to mediate neuronal functions in synaptic plasticity. We describe a new pathway integrating the highly conserved tyrosine residue in the cytoplasmic tail of MHC-I in mediating AMPA receptor expression and intracellular signalling via outside-in or reverse signaling. These findings reinforce the importance of a single amino acid on the MHC-I cytoplasmic domain in regulating excitatory neurotransmission in the CNS, opening the door towards modulating MHC-I expression and signalling to modify the function of excitatory synapse function that is intimately connected to learning and memory.

## Materials and methods

### Experimental animals

The study has been reported in accordance with ARRIVE guidelines^62^ and all mouse experiments were approved by the Animal Care Committee at UBC. We have previously described C3H/He mice carrying the tyrosine mutation on its cytoplasmic tail (ΔYK^b^C3H) or expressing transgenic WT MHC-I allele H-2K^b^ (WTK^b^C3H)^23^. We crossed H2K^b^2D^b^ double knockout (H2K^−/−^D^−/−^) mice with the WTK^b^C3H and ΔYK^b^C3H strains, respectively to generate the WTK^b^ H2D^−/−^ and ΔYK^b^ H2D^b−/−^ strains. WTK^b^C3H and ΔYK^b^C3H strains were originally maintained on a C3H/He background and express MHC-I genes H2K^k^ and H2D^k^ as well as the knocked-in transgenic H-2K^b^ gene (ΔYK^b^ or WTK^b^). The mice were backcrossed onto an H2K^−/−^D^−/−^ background (H2K^b^2D^b^ double knockout) for at least 10 generations while the original line was retired. The progeny of the crosses were genotyped by PCR and phenotyped by flow cytometry to establish the presence of MHC-I alleles H2K^k^, K^b^, D^k^ and D^b^ as well as the disrupted H-2K^b^ and D^b^ genes. PCR to detect presence of knocked in transgenic H-2K^b^ (ΔYK^b^ or WTK^b^) used H-2K^b^ specific oligonucleotides, 5′-TCGCTGAGGTATTTCGTC-3′ and 5′-TTGCCCTTGGCTTTCTG T-3′^63–65^.

Western blotting and kinase array studies were performed on six-month-old mice. Mice were kept under a 12-h light/dark cycle and fed standard lab chow and water ad libitum. All animal experiments were performed at the University of British Columbia, Vancouver, BC, Canada and were conducted in compliance with the University of British Columbia Animal Care Committee under the direction of the Canadian Council for Animal Care.

### Western blot

Mouse hippocampi were dissected from WT and ΔY mice (n = 3) and were immediately snap-frozen and stored at − 80 °C until processed. Hippocampi were homogenized in 2 mL of homogenization buffer (0.32 M sucrose, 1 mM ethylenediaminetetraacetic acid, 1 mg/mL bovine serum albumin, 5 mM 4-(2-hydroxyethyl)-1-piperazineethanesulfonic acid, pH 7.4) using a Dounce homogenizer and immediately mixed 3:5 with 2 × sodium dodecyl sulphate (SDS) sample buffer. Homogenates were separated by SDS polyacrylamide gel electrophoresis followed by transfer to a nitrocellulose membrane (Thermo Scientific, Rockford, IL, USA) as described previously^66^ and were done on 4–15% gradient gel. The primary antibodies used were 1:1000 dilutions of mouse anti-PSD95 (Cat# 75-028, UC Davis/NIH NeuroMab Facility, Davis, CA, USA), mouse anti-GluN2B/NR2B (Cat# 75-101, UC Davis/NIH NeuroMab Facility), goat anti-actin (Cat# sc-1615, Santa Cruz Biotechnology, Santa Cruz, CA, USA) and rabbit anti-GluA2/3 (Cat# AB1506, Millipore, Temecula, CA, USA), mouse primary anti-p38 alpha/MAPK14 antibody (Cat# ab170099 Abcam, Cambridge, UK), anti-p38 (phospho T180 + Y182) antibody (Cat# ab45381 Abcam, Cambridge, UK) and anti-alpha Tubulin antibody (Cat# ab7291 Abcam, Cambridge, UK). The secondary antibodies were 1:10,000 dilutions of AlexaFluor-680 conjugated donkey anti-mouse IgG H + L (Cat# A10038, Life Technologies, Eugene, OR, USA), AlexaFluor-680 conjugated donkey anti-rabbit (Cat# A10043, Life Technologies) and AlexaFluor 790 conjugated donkey anti-goat IgG H + L (Cat# A11370, Life Technologies). Fluorescence was detected and quantitated using Odyssey Infrared Imager (LI-COR, Lincoln, NE, USA). Signal intensities were normalized against β-actin  or alpha-tubulin. Results are shown as mean ± SEM.

### Phospho-kinase array

Protein kinase phosphorylation levels were measured in hippocampus homogenates (see “Western blot” section for preparation) from ΔY and WT mice using the Proteome Profiler Human Phospho-Kinase Array kit (ARY003B; R&D SYSTEMS, Minneapolis, MN, USA) according to manufacturer's instructions. The assay is a nitrocellulose membrane-based sandwich ELISA, which has captured antibodies spotted in duplicates on the membrane. The total protein concentration of hippocampal homogenates was measured by Bradford protein assay, and 300 μg of total protein was loaded on the nitrocellulose membrane. The signal intensity from the nitrocellulose membrane, which is proportional to the phosphorylation level of the bound analyte, was measured with the Protein Array Analyzer plugin for ImageJ (NIH, Bethesda, MD, USA). The signal intensity of ΔY samples was normalized to WT, and a heat map was generated depicting the change in phosphorylation intensities using GraphPad Software7, Inc. (La Jolla, CA, USA). Results were verified by Western blot using an antibody against tyrosine-protein kinase p38. As a control for the kinase assay, we performed Western blot analysis on p38 since p38 MAP kinase pathway dysregulation of MHC-I-expressing monocytic cells correlates with MHC-I misfolding^27^. The antibodies used were an anti-p38 alpha/MAPK14 antibody, anti-p38 (phospho T180 + Y182) antibody and anti-alpha Tubulin antibody. Secondary antibodies: Goat Anti-Mouse IgG H&L (HRP) preabsorbed (ab97040) and Invitrogen. Goat anti-Rabbit IgG (H + L) Secondary Antibody, HRP (31460).

### Statistical analysis

Unless otherwise stated, all experiments were repeated three times, and the means and statistical significance were determined. The data was then analyzed using the Psych package in RStudio (R version 4.2.0). The resulting summary statistics were used to assess the skewness and kurtosis of data distribution. Shapiro–Wilk and Kolmogorov–Smirnoff tests were performed in R to measure the parameters of normal distributions. Normally distributed data were subjected to the appropriate *t* test (unpaired or paired) statistical test (both using GraphPad Prism software, version 9.4.1) with 95% confidence intervals. P values less than 0.05 were considered significant. All data were represented as mean ± SEM.

### Supplementary Information

Supplementary Figures.

## Data Availability

The datasets used and/or analyzed during the current study are available from the corresponding author upon reasonable request.

## Abbreviations

ΔY: Tyrosine 321 to phenylalanine (Y321F)
AMPA: α-Amino-3-hydroxy-5-methyl-4-isoxazolepropionic acid
CA1: *Cornu ammonis* 1
CNS: Central nervous system
CREB: Cyclic AMP-responsive element-binding protein
EGFR: Epidermal growth factor receptor
HD: Head diameter
MHC-I: Major histocompatibility complex class I
mTOR: Mechanistic/mammalian target of rapamycin
NMDA: *N*-Methyl-d-aspartate
PBS: Phosphate-buffered saline
PSD: Post-synaptic density
WT: Wild type
