# Supplementary material for: Outside-in signaling through the major histocompatibility complex class-I cytoplasmic tail modulates glutamate receptor expression in neurons
Source: Sci Rep. 2023 Aug 11;13:13079. doi: 10.1038/s41598-023-38663-z (PMC10421907; doi:10.1038/s41598-023-38663-z)
Supplement: Supplementary file 1 — Supplementary Figures. [file 41598_2023_38663_MOESM1_ESM.pdf]

Supplementary Information

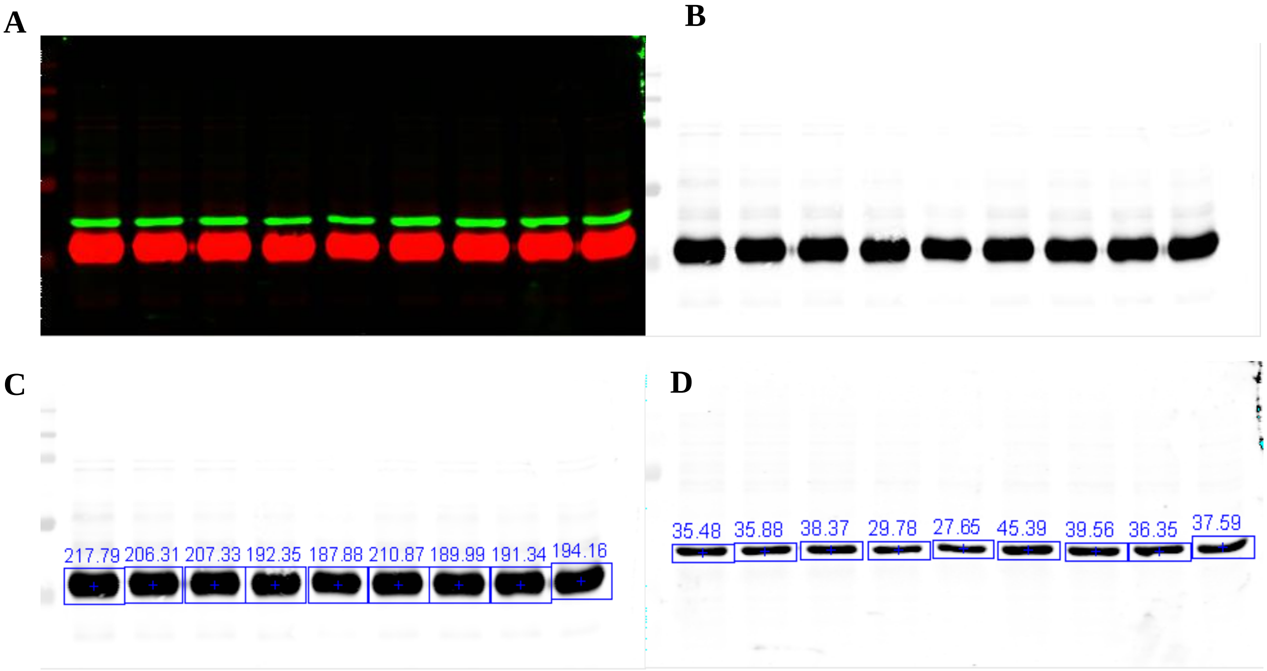

Supplementary Figure 1: *Full Western Blot image of A) Synaptophysin – Actin. Raw Image B) Synaptophysin only C) Synaptophysin only – Quantitated and D) Actin only (paired with synaptophysin) – Quantitated*

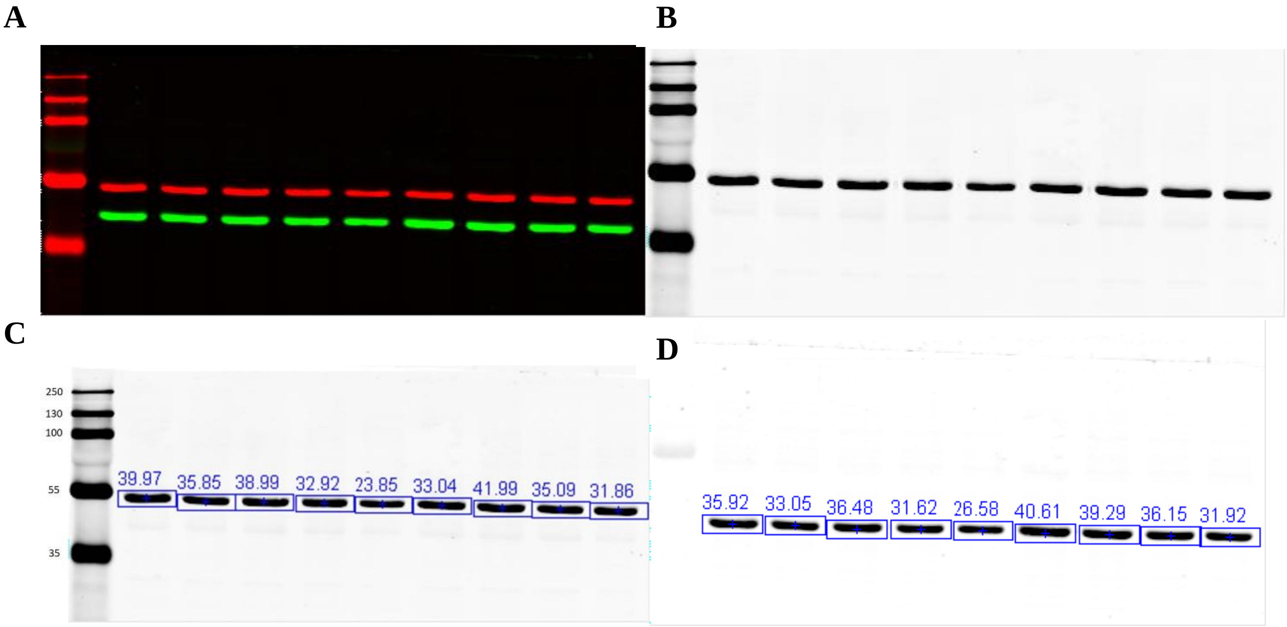

Supplementary Figure 2: *Full Western Blot image of A) VGlut1 and Actin. Raw Image B) VGlut1 only C) VGlut1 only – Quantitated and D) Actin only (paired with VGlut1) – Quantitated*

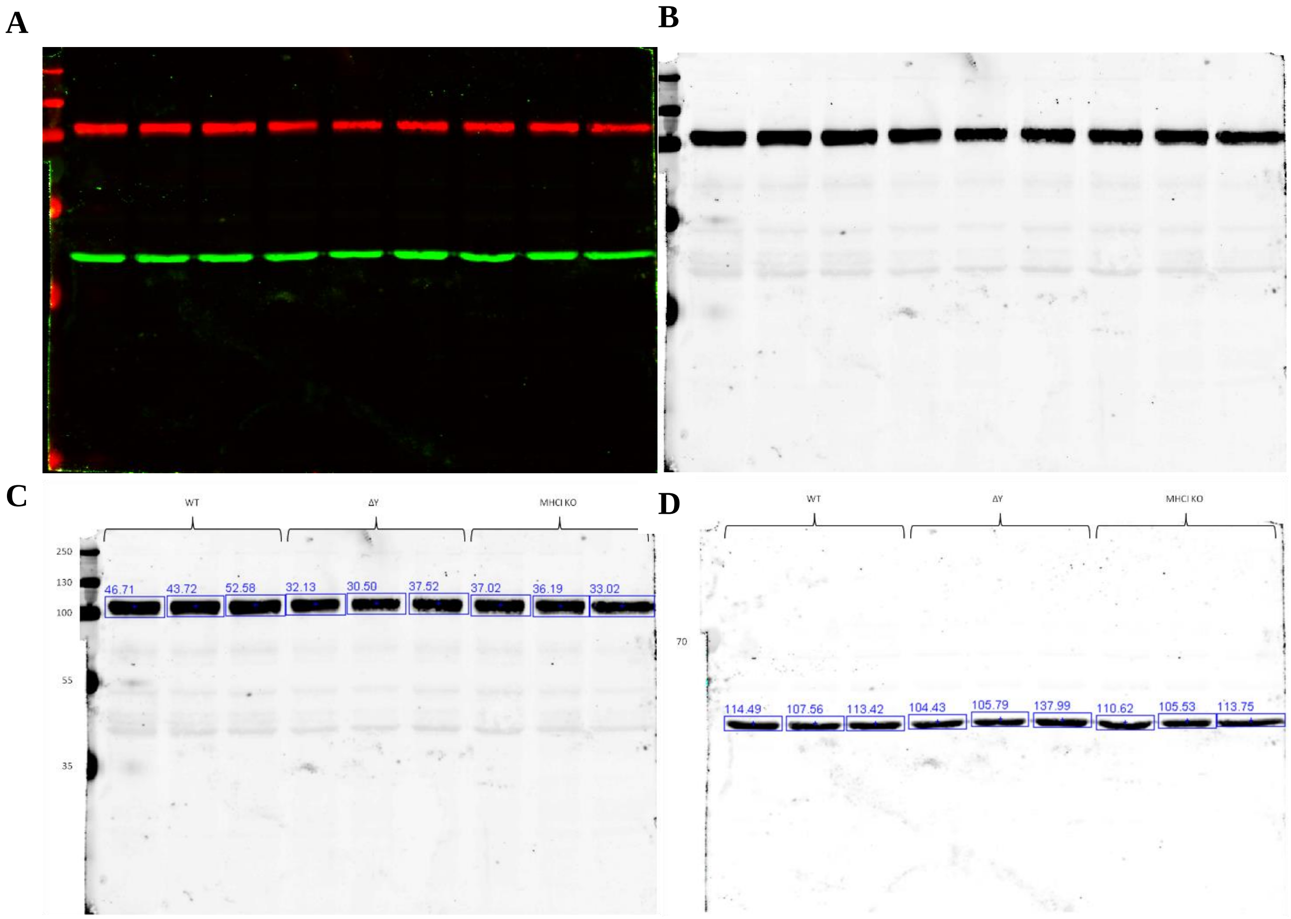

Supplementary Figure 3: *Full Western Blot image of A) GluA2/B and Actin. Raw Image B) GluA2/B C) GluA2/3 only – Quantitated and D) Actin only – Quantitated*

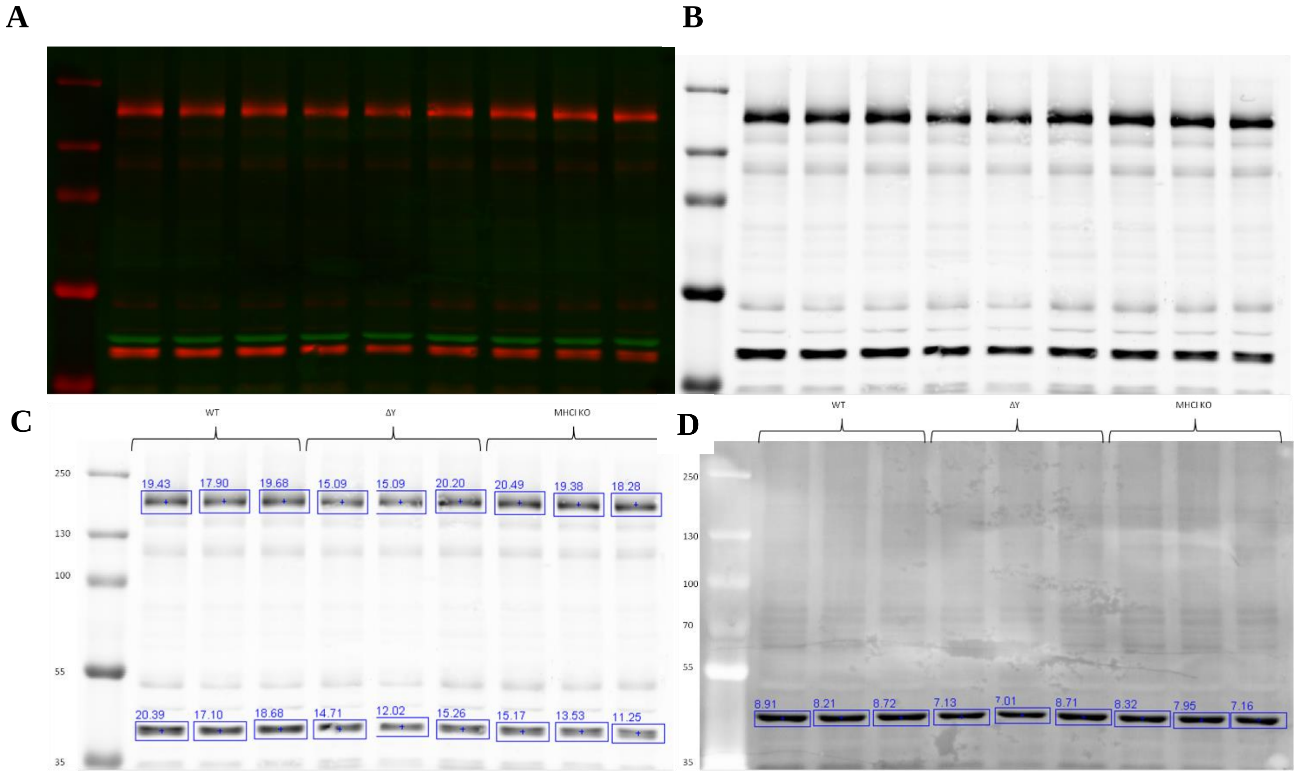

Supplementary Figure 4: *Full Western Blot image of A) GluN2B and Actin. Raw image B) GluN2B C) GluN2B only – Quantitated and D) Actin only – Quantitated*

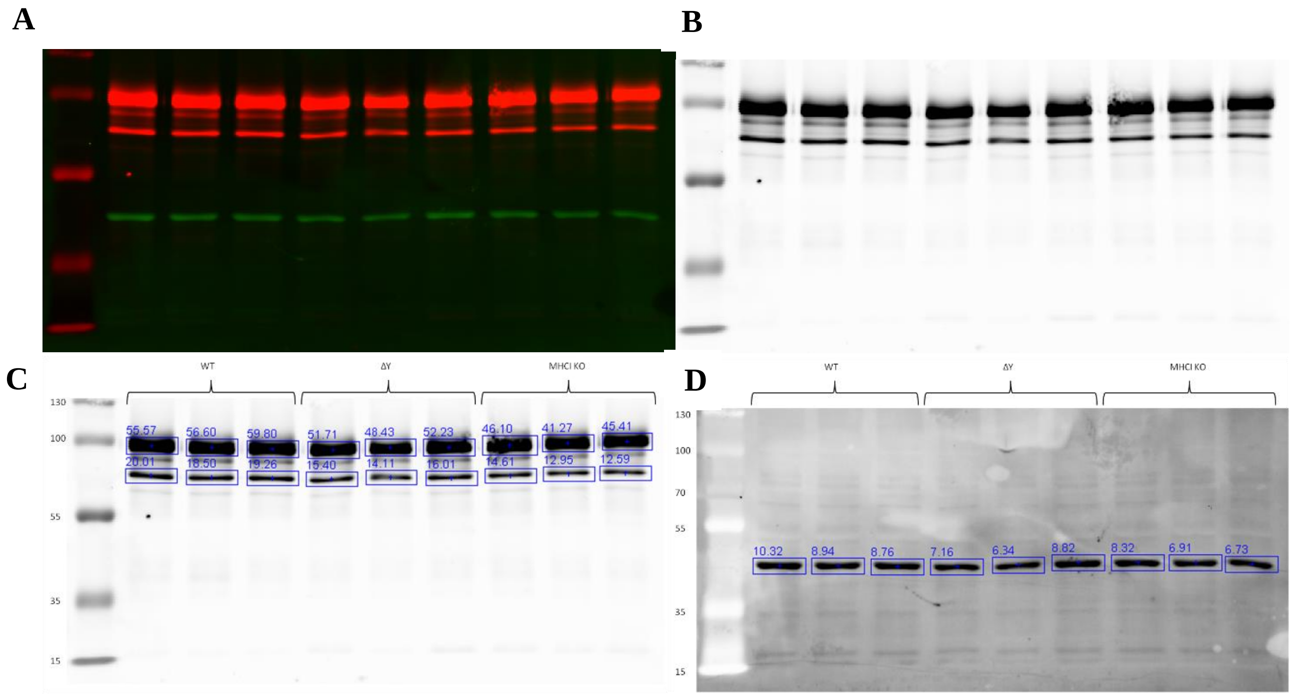

Supplementary Figure 5: *Full Western Blot image of A) PSD95 and Actin. Raw image B) PSD95 C) PSD95 only – Quantitated and D) Actin only – Quantitated*

A

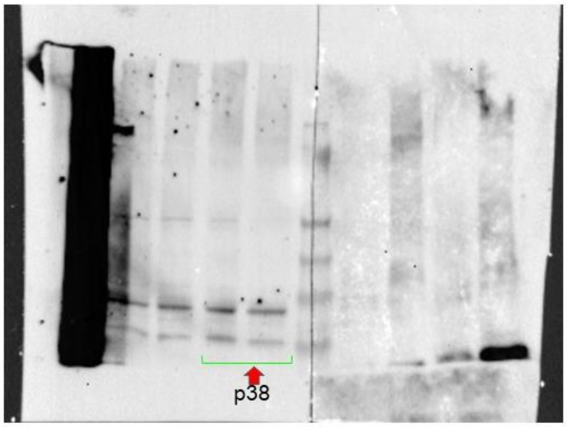

B

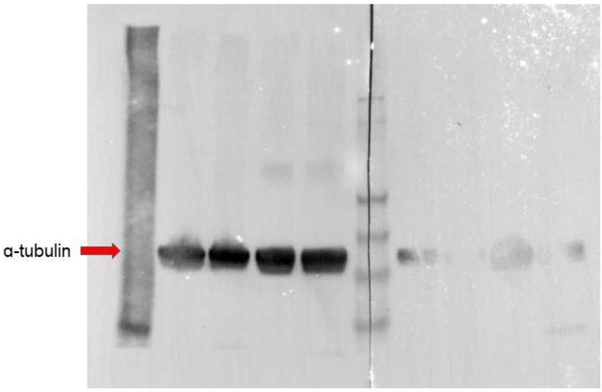

C

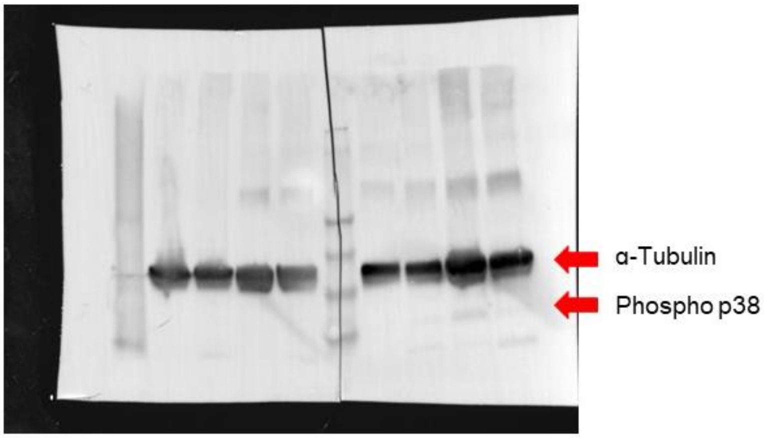

D

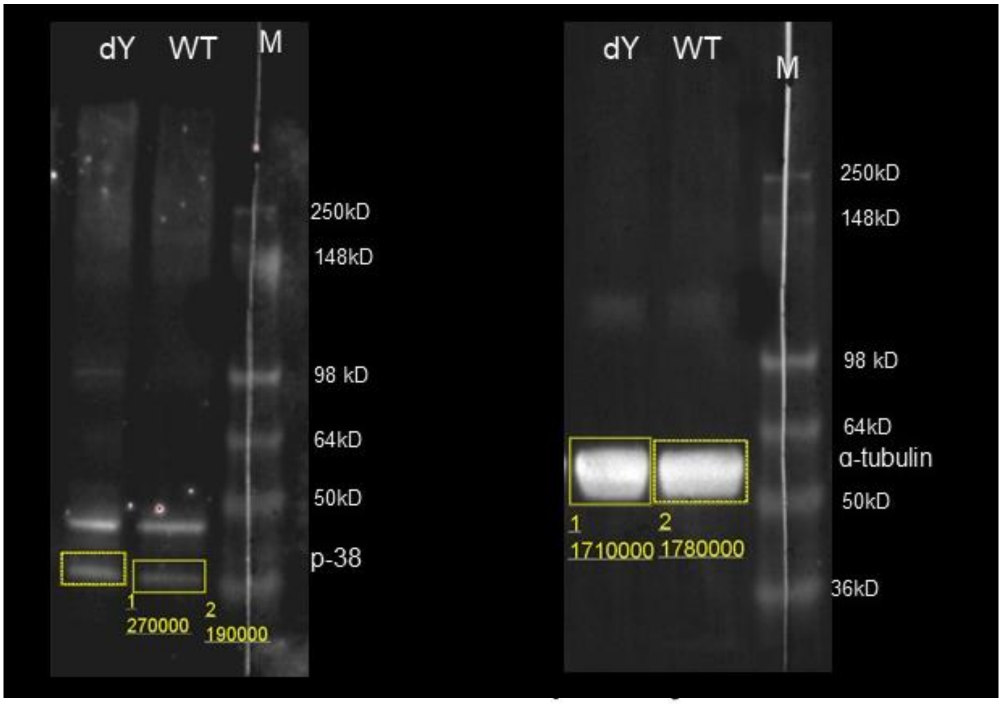

E

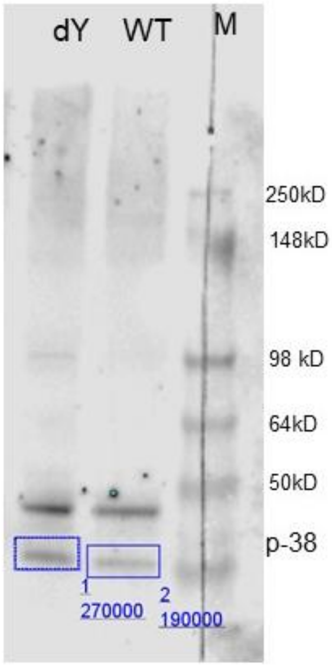

F

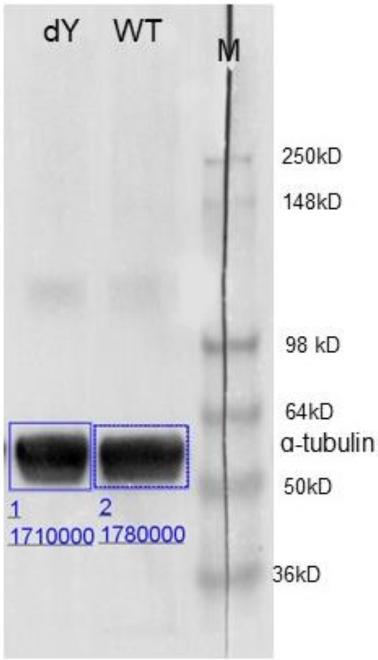

Supplementary Figure 6: *Western Blot image of A) Full – p38 B) Full – a-tubulin C) Full – phosphor p-38 D) P38 and a-tubulin. Raw image E) P38 only – Quantified and F) a-tubulin only – Quantified*

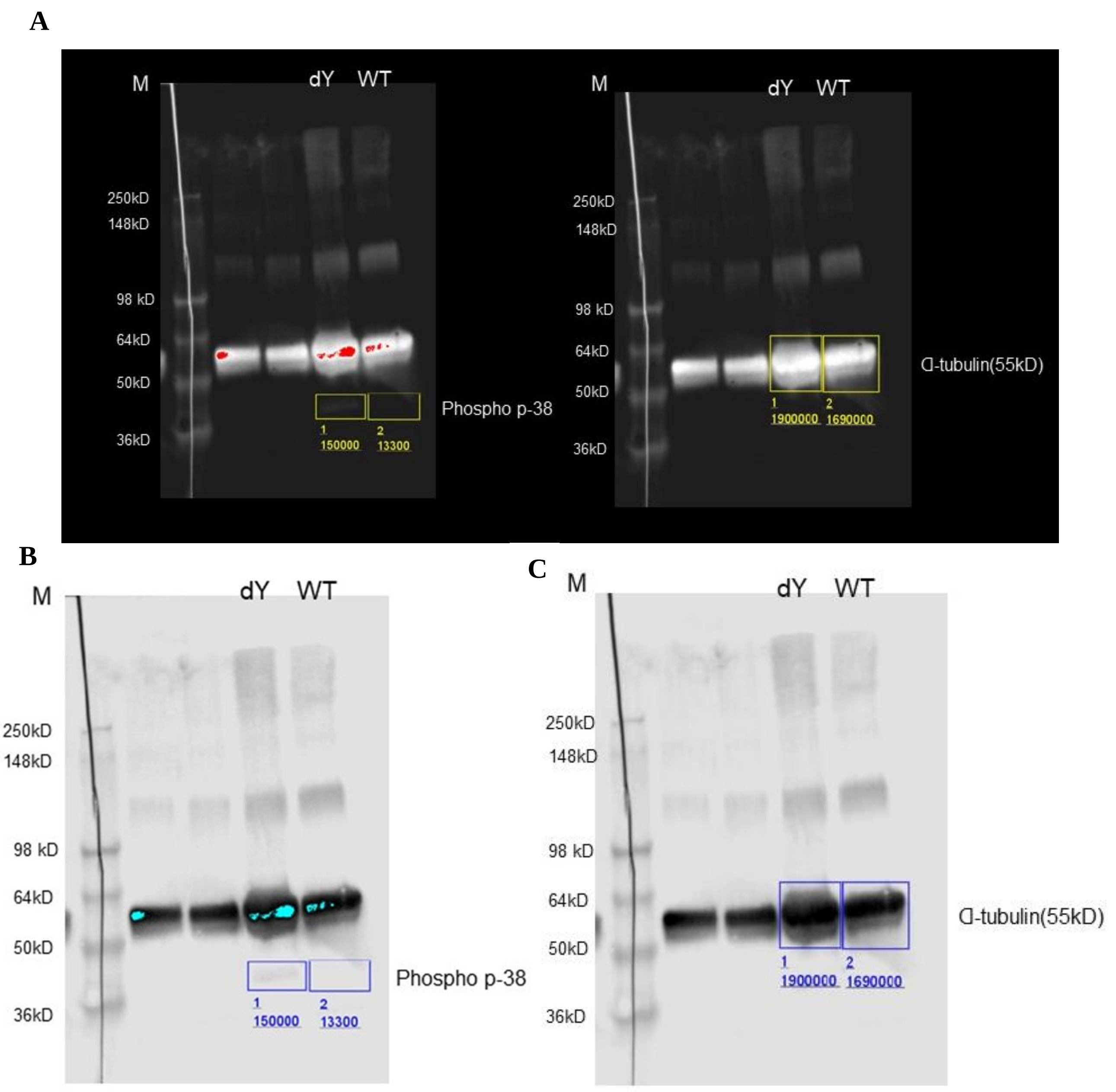

Supplementary Figure 7: Western Blot image of A) Phospho p-38 and a-tubulin. Raw image B) phosphor p-38 only – Quantified and C) a-tubulin only – Quantified
